# The Effects of Testosterone on Oxidative Stress Markers in Mice with Spinal Cord Injuries

**DOI:** 10.22074/ijfs.2016.4773

**Published:** 2016-04-05

**Authors:** Hamid Choobineh, Mohammad Ali Sadighi Gilani, Parvin Pasalar, Issa Jahanzad, Rostam Ghorbani, Gholamreza Hassanzadeh

**Affiliations:** 1 Department of Anatomy, School of Medicine, Tehran University of Medical Sciences, Tehran, Iran; 2 School of Allied Medical Sciences, Tehran University of Medical Sciences, Tehran, Iran; 3 Zoonosis Research Center, Tehran University of Medical Sciences, Tehran, Iran; 4 Department of Urology, School of Medicine, Tehran University of Medical Sciences, Tehran, Iran; 5 Department of Biochemistry, School of Medicine, Tehran University of Medical Sciences, Tehran, Iran; 6 Department of Pathology, School of Medicine, Tehran University of Medical Sciences, Tehran, Iran; 7 Department of Anatomy, School of Medicine, Kermanshah University of Medical Science, Kermanshah, Iran

**Keywords:** Spinal Cord Injury, Infertility, Testosterone, Oxidative Stress, Reactive Oxygen Species

## Abstract

**Background:**

Spinal cord injury (SCI) causes infertility in male patients through erectile dysfunction, ejaculatory dysfunction, semen and hormone abnormalities. Oxidative stress (OS) is involved in poor semen quality and subsequent infertility in males with SCI. The aim of this study is to examine the effects of SCI on the level of testosterone hormone.

**Materials and Methods:**

In this experimental study, we evaluated the effects of exogenous testosterone on the activity of the antioxidant enzymes superoxide dismutase (SOD) and glutathione peroxidase (GPx) as well as the levels of malondialdehyde (MDA) and protein carbonylation (PCO), as markers of OS, in 10 groups of SCI mice. Total antioxidant capacity (TAC) was determined using the 2,29-azinobis-(3-ethylbenzothiazoline- 6-sulfonic acid) (ABTS) radical cation assay.

**Results:**

Exogenous testosterone administration in mice with SCI significantly reduced SOD and GPx enzyme activities and MDA level. There was no significant decrease in PCO content. In addition, TAC remarkably increased in the sham and SCI groups not treated with testosterone but remained unchanged in all other experimental groups. Exogenous testosterone also reduced serum testosterone levels in all groups except the positive control group.

**Conclusion:**

Our cumulative data indicated that SCI could cause sterility by disturbing the plasmatic testosterone balance. The normal level of endogenous testosterone was not completely restored by exogenous testosterone administration.

## Introduction

Spinal cord injury (SCI) is a traumatic or nontraumatic injury which occurs most often in 16-45 year-old males at the peak of their reproductive lives, permanently affecting quality of life (1). The majority of male patients with SCI have a weak reproductive function and distinct sperm profile with normal sperm count, but the sperm motility is abnormally low (1,4). However, the mechanisms responsible for poor sperm quality in men with SCI have not been clearly defined. It has been reported that abnormalities of hormones and hypothalamic-pituitary-gonadal axis dysfunction can be involved as a consequence of SCI in males (5). On the other hand, there is increasing evidence for the impact of oxidative stress (OS) on the sperm quality in this group of patients (2). Several studies have demonstrated a significant increase in the generation of reactive oxygen species (ROS) in males with SCI. It is well established that physiological level of ROS is necessary for normal sperm function and reactions that include oocyte fusion, capacitation and acrosome reaction (AR) while the excess amounts of ROS in semen can induce OS which negatively affect spermatozoa (2,6). Seminal ROS strike a wide range of essential biomolecules such as proteins, lipids, carbohydrates and nucleic acids, and affect their functions. This impact may consequently be involved in DNA damage, decreased sperm motility, reduced sperm viability, sperm dysfunction, and semen hyperviscosity (7). Lipid peroxidation of sperm plasma membranes by ROS causes reduced membrane fluidity (1,7). 

Seminal fluid contains several defense mechanisms which are focused on oxidant scavenging to protect spermatozoa from detrimental oxidative injury. These include important antioxidant enzymes such as catalase, superoxide dismutase (SOD) and glutathione peroxidase (GPx) which quench hydrogen peroxide and the excess free superoxide radicals. The seminal fluid also contains non-enzymatic antioxidants such as ascorbic acid (vitamin C), α-tocopherol (vitamin E), carnitine and pyruvate. In this regard, elevated OS and reduced antioxidant activity in the seminal plasma lead to damaged sperm function and subsequent male infertility. It has been reported that infertile patients with and/or without SCI have discrepancies in seminal levels of ROS (7,9). Imbalanced hormonal levels, especially testosterone and follicle-stimulating hormone (FSH), are observed in SCI patients (2,7). There are conflicting reports that demonstrate the direct roles of testosterone and FSH hormones in reproductive dysfunction in men with SCI. 

Therefore, the present study aimed to examine the effects of SCI on the level of testosterone hormone. We determined whether a testosterone imbalance was involved in increased OS and resultant reproductive dysfunction. 

## Materials and Methods

### Animals

Adult male mice, 4 to 6 months of age, that weighed 15 to 25 g were obtained from the School of Pharmacy, Tehran University of Medical Sciences, Tehran, Iran. Mice were kept in temperature-controlled quarters on a 12:12 hour light:dark schedule, with standard mouse pellets and drinking water ad libitum.

### Experimental design

In this experimental, we randomly divided the mice into 10 groups each, with 6 animals per group according to Table 1.

**Table 1:** Experimental design


	Laminectomy	SCI	Testosterone injection	Sampling day after SCI

P. Control	-	-	0.1 mg/kg BW-7 days	After completion of the period of injection
N. Control	-	-	-	Coincident with P. Control
SCI-0.0-7	+	+	-	1 week
SCI-0.0-35	+	+	-	35 days
SCI-0.1-7	+	+	0.1 mg/kg BW-7 days	2 weeks
SCI-0.1-14	+	-	0.1 mg/kg BW-7 days	3 weeks
SCI-0.1-35	+	+	0.1 mg/kg BW-35 days	35 days
SCI-0.1-42	+	+	0.1 mg/kg BW-35 days	42 days
P. Laminectomy(Sham group 1)	+	-	0.1 mg/kg BW-7 days	2 weeks after laminectomy
N. Laminectomy(Sham group 2)	+	-	-	Coincident with P. Laminectomy group


SCI; Spinal cord injury and BW; Body weight.

SCI-0.1-7 and SCI-0.1-42 received injections of testosterone one week after infliction of the SCI. SCI-0.1-14 received an injection of testosterone two weeks after infliction of the SCI. SCI-0.1-35 received an injection of testosterone at the same time as infliction of the SCI. N. Laminectomy was a sham group that did not receive any testosterone and P. Laminectomy was a sham group that received a testosterone injection.The N. Laminectomy and P. Laminectomy groups each underwent a sham operation. The N. Laminectomy did not receive testosterone replacement whereas the P. Laminectomy group received testosterone replacement.

### Experimental protocol

We performed the SCI according to procedures by Yu et al. (10). We used a model that provided extradural compression of the spinal cord from the dorsal side. Briefly, the animals were anesthetized by xylazine [5 mg/kg body weight (BW)] and ketamine (50 mg/kg BW) injections; the laminae of the T9 and T11 vertebrae were removed, leaving the dura intact. The animals were placed in the prone position in a stereotaxic apparatus to perform the laminectomy and stabilize the spinal cord. A weight of 35 g was applied onto the intact dura for 5 minutes, using a curved rectangular plate (2.2×5 mm). According to evidence, serum testosterone levels have been shown to significantly reduce 3-7 days after SCI. The serum level of testosterone might return to normal levels by 14 days due to cellular compensatory mechanisms (11). The duration of spermatogenesis in mice is 32-35 days. Accordingly, we have divided the mice into six SCI groups, without and with testosterone administration, according to different intervals of administration and times for blood sampling (7, 14, 35 and 42 days).

Plasma levels of testosterone were estimated by a commercial enzyme linked immunosorbent assay (ELISA) kit (Cayman Chemical, USA) and expressed as ng/ml. The malondialdehyde (MDA, Hitachi, Japan) component of the blood samples was expressed as nanomoles of MDA created per mg protein and measured by the spectrophotometric method. We measured total antioxidant capacity (TAC) of plasma by the ABTS radical cation assay (12). SOD was examined by a Biovision kit (Biovision, USA). GPx was measured with a Randox kit (Randox, UK). Protein carbonylation content of mouse serum was assayed using a BioCell Protein Carbonyl Assay Kit (BioCell, New Zealand).

### Statistical analysis

All data were expressed as mean ± SEM of three independent experiments. The data were analyzed by one-way ANOVA followed by the Tukey test. The statistical analyses were performed using Graphpad Prism statistical software (9). P<0.05 was regarded as statistically significant.

## Results

During the 6 weeks of testosterone treatment, all of the mice groups remained healthy and grew at an ordinary rate. There was no significant difference between the body weight and weights of the testes, epididymis, seminal vesicles or ventral prostate of mice treated with testosterone or sham (data not shown).

**Fig.1:**
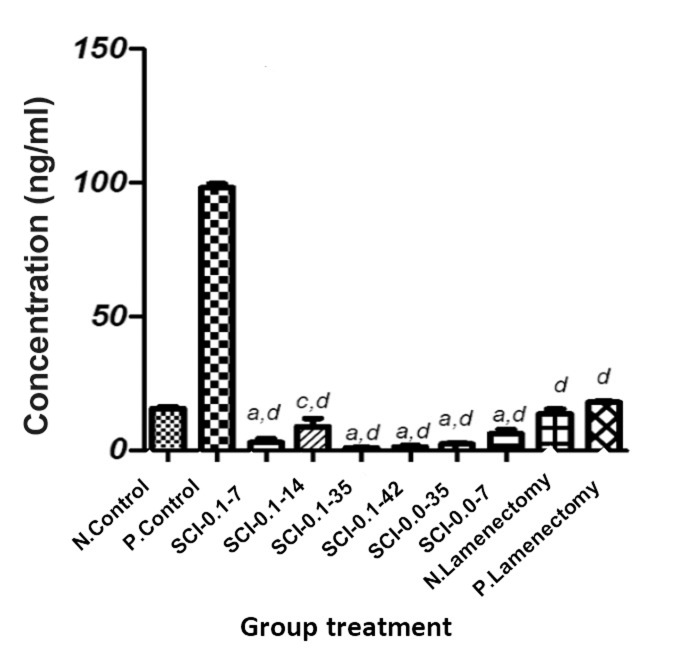
Effect of exogenous testosterone on plasma testosterone levels. The details of groups is in material and method section. Results are expressed as mean ± SD. a; P<0.001 compared with N. Control, c; P<0.05 compared with N. Control and d; P<0.001 compared with P. Control.

The results presented in Figure 1 revealed that administration of exogenous testosterone led to a significant (P<0.001) reduction in total plasma testosterone level in all groups except for the positive control. However, the sham groups exhibited relatively less decrease compared with the other groups.

**Fig.2:**
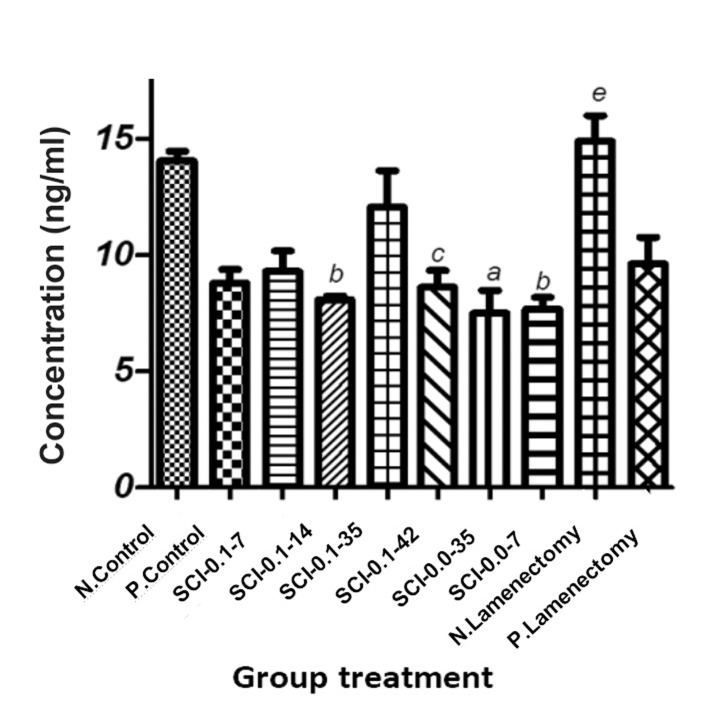
Effect of exogenous testosterone on malondialdehyde (MDA) level. The details of groups is in material and method section. Results are expressed as mean ± SD. a; P<0.001 compared with N. Control, b; P<0.01 compared with N. Control, c; P<0.05 compared with N. Control and e; P<0.01 compared with P. Control.

As shown in Figure 2, MDA levels decreased (significantly and insignificantly) in mice plasma as a consequence of testosterone administration while the negative sham group had a significantly increased MDA level (P<0.01).

The influence of testosterone on other antioxidant biomarkers such as GPx, SOD, protein carbonyl and TAC are presented in Table 2. The findings depicted that testosterone administration had various effects on GPx and SOD in the tested groups. The results showed that testosterone administration significantly reduced GPx activity in the SCI-0.0-7 group relative to the control group. There was significantly diminished GPx activity in the sham groups with testosterone administration. On the other hand, the testosterone administration did not significantly affect GPx. In contrast, the testosterone treatment showed a considerable increase in GPx activity in the SCI group with administration of testosterone (0.1 mg/kg of body weight) after one week compared to the positive control group (P<0.001). There was a similar trend observed for SOD activity. According to Table 2, the testosterone administration showed fluctuations on SOD activity, which in some cases caused significantly increased (P<0.001 compared with both negative and positive groups) SOD activity in the positive sham group. However, in some tested groups, the administration of testosterone led to a nonsignificant reduction of SOD activity. In contrast, the effect of testosterone administration on TAC did not show considerable differences in the tested groups. We observed a significant increase in the sham (P<0.001) and SCI groups without testosterone treatment (P<0.05) in TAC plasma content in comparison with both positive and negative control groups. In addition, all tested groups showed a significant reduction (P<0.001) in protein carbonyl levels relative to the positive control, but not the negative control. The SCI groups without testosterone treatment showed considerably elevated (P<0.001 and P<0.01) protein carbonyl levels in comparison to the negative control. Generally, testosterone treatment failed to reduce protein carbonyl levels in all tested subjects.

**Table 2:** Effect of exogenous testosterone on testicular antioxidant enzymes (GPx and SOD)


	N. Control	P. Control	SCI-0.1-7	SCI-0.1-14	SCI-0.1-35	SCI-0.1-42	SCI-0.0-35	SCI-0.0-7	N. Lamenectomy	P. Lamenectomy

**GPX**	27.98 ± 1.64	31 ± 2.89	36.43 ± 8.41^d^	27 ± 2.60	33.5 ± 1.87	27.6 ± 3.13	24.02 ± 0.52^f^	22.5 ± 1.20^d^	28.1 ± 3.32	18.3 ± 2.40^a, d^
**SOD**	326.5 ± 183.20	276.7 ± 52.03	310.8 ± 101.90	319.7 ± 26.76	147.2 ± 21.14	292.2 ± 78.87	449.3 ± 27.51	308 ± 103.70	392.8 ± 46.86	619.2 ± 124.20^a, d^
**Protein Carbonyl**	0.28 ± 0.013	0.38 ± 0.051	0.30 ± 0.031^d^	0.27 ± 0.013^d^	0.29 ± 0.010^d^	0.28 ± 0.029^d^	0.36 ± 0.009^a^	0.35 ± 0.030^b^	0.26 ± 0.004^d^	0.28 ± 0.006^d^
**TAC**	2.11 ± 0.0	2.11 ± 0.0	2.11 ± 0.0	2.11 ± 0.0	2.11 ± 0.0	2.027 ± 0.16	2.955 ± 0.12	3.19 ± 0.31	2.792 ± 0.05	2.858 ± 0.12


SOD; Superoxide dismutase, GPx; Glutathione peroxidase, SCI; Spinal cord injury, TAC; Total antioxidant capacity, ^a^; P<0.001 compared with N. Control, ^b^; P<0.01 compared with N. Control, ^d^; P<0.001 compared with P. Control, and ^f^; P<0.05 compared with P. Control.

## Discussion

Recently, attention has been paid to male sterility after SCI. Most male patients with SCI have poor semen quality, as evidenced by leukocytospermia and sperm motility (3, 4, 7, 13, 14). The presence of activated T cells in men with SCI leads to cytokine synthesis, which in turn affects human sperm motility and increases the production of ROS (15). It has been reported that SCI temporary and intensely influences the pituitarytesticular hormone axis. These alterations may be involved in Sertoli cell dysfunctions and consequent abnormalities in regular spermatogenesis (11). However, the cause of infertility in men with SCI is not clearly recognized. It has been reported that infertility might be related to sexual hormone imbalance and/or ROS production (2, 6). Therefore, elucidation of some of the differences in the literature might be essential

In this regard, we evaluated whether SCI caused sexual hormone imbalances and ROS production. We also examined whether exogenous testosterone could make tribulation. Our results showed that the normal level of plasma testosterone was not restored by exogenous testosterone administration in all SCI mice except for the SCI 0.1 (2 W) groups. In agreement with a number of studies, our observations indicated that the administration of exogenous testosterone led to downregulation of natural testosterone production by the testes (16). Since such a decrease occurred only in the spinal cordoperated mice, and not in sham-operated ones that endured similar surgery-related stress.

It could be concluded that SCI might at least partially be involved in testosterone downregulation. However, the trivial immobilization of the mice following SCI might have resulted in more stress, which could have influenced the pituitarytesticular hormone axis and consequently suppressed natural testosterone production (11). It has also been reported that the denervation of testis in immature rats resulted in impairment of Leydig cell androgen production, which was comparable to the subjects with SCI damage (17).

The acute suppression of testosterone production in testes after SCI, with or without an attendant decline in serum FSH levels, could definitely compromise Sertoli cell functions and cause some of the abnormalities in spermatogenesis (11). These abnormalities might be attributed to increased OS that has resulted from an imbalance between the production of ROS and antioxidant agents (18). ROS, unstable and extremely reactive by-products of normal metabolism, mediate oxidative damages to cellular macromolecules (9). Since testosterone typically improves the metabolic rate (19, 20), it can be expected that a high dose of testosterone level may be involved in the imbalance between ROS production and antioxidant defenses causing an increased risk of OS. In this regard, several researchers have reported that testosterone plays a pro-oxidant role and induces OS in mammalian tissues (20). On the other hand, it has also been reported that testosterone has an antioxidant effect in the human prostate (21) and rat nervous system (9). These outcomes consequently indicate that the pro-oxidant property of testosterone is tissue and sex-dependent. In this regard, testes are principally susceptible to ROS-induced injury by virtue of testosterone’s pro-oxidant activity.

Inconsistently, Chainy et al. (6) have reported that elevated MDA levels in response to testosterone treatment, whereas Peltola et al. (22) reported that testosterone decreased the level of MDA. In an *in vitro* study, Mooradian showed that administration of exogenous testosterone did not have significant pro-oxidant activity. Our results indicated that the MDA level did not increase, but reduced in groups that received testosterone, which suggested that testosterone suppressed H_2_O_2_ production and decreased production of MDA (23).

Protein carbonyl, as a marker of OS, is one of the OS by-products which form during the interaction between ROS and proteins. This interaction modifies biomolecules and changes their functions eventually leading to irretrievable cellular damage (24). In agreement with some studies, our results (SCI-operated without testosterone administration) have demonstrated that protein carbonyl increases while the level of plasma testosterone decreases (25). However, administration of exogenous testosterone showed no significant effect on protein carbonyl production in the SCI groups relative to the negative control. Therefore, this suggested that exogenous testosterone did not have a protective effect on this type of oxidative damage.

Plasmatic TAC is the other antioxidant defense system against OS. The functional sum of antioxidants in plasma is used as measure of extracellular antioxidant barrier (26). Thus, we have measured TAC after testosterone administration in SCI-operated groups. Our findings indicated that administration of exogenous testosterone did not increase plasma TAC. However, TAC levels increased in the sham group and groups that did not receive testosterone. Therefore, our results reinforced the pro-oxidative properties of testosterone. Contrary to our results, Mancini et al. (26) reported that TAC had a significant association with total testosterone in male subjects. Such a discrepancy might be attributed to several factors such as gender-specific gene expression, vascular factors, distribution of body fat, and adaptation to aging. In addition, studies on exogenous testosterone administration were affected by dose, duration and route of administration. Thus, a study of antioxidant regulation by steroids could help to elucidate molecular mechanisms of testosterone function.

Injections of testosterone into adult mice manipulated the level of testosterone in the testes. Our results revealed that the testosterone injection caused antioxidant enzymes (GPx and SOD) reduction in testes in most groups, which agreed with findings reported by Chainy et al. (6). The precise mechanism of testosterone-induced reduction in the levels of GPx and SOD enzymes in the testis was not well reported. It has been reported that administration of 10 mg testosterone to intact rats caused a profound reduction (82%) in the serum luteinizing hormone (LH) level with no alteration in the FSH level (6) which regulated testosterone production in the testis (27). In addition, these antioxidant enzymes have synergistic functions. An abnormality of one of the antioxidant enzymes can affect the activities of the other enzymes. A reduction in SOD activity causes an elevation in the level of O_2-_, which in turn causes inactivation of catalase (CAT) activity. Equally, when GPx or CAT fails to eradicate H_2_O_2_, the content of H_2_O_2_ may be upregulated by inactivation of SOD and vice versa (6). In general, administration of exogenous testosterone causes antioxidant enzyme reduction followed by OS induction.

## Conclusion

Collectively, SCI, a neurogenic impairment, causes infertility through disturbing the plasma testosterone balance which could not be retrieved by administration of exogenous testosterone. In this regard, SCI led to a slight change in oxidative markers, with the exception of MDA which decreased. There was reduced free radical scavenging activities of SOD and GPx. Such an effect reinforced the pro-oxidant property of testosterone. Therefore, administration of exogenous testosterone would not compensate sexual hormone disturbance along with anti-oxidative protective effects. However, the exact causal mechanism leading to sexual hormone disturbances in males with SCI remains to be elucidated.
